# *Clostridium perfringens* Beta-Toxin Induces Necrostatin-Inhibitable, Calpain-Dependent Necrosis in Primary Porcine Endothelial Cells

**DOI:** 10.1371/journal.pone.0064644

**Published:** 2013-05-29

**Authors:** Delphine Autheman, Marianne Wyder, Michel Popoff, Katharina D’Herde, Stephan Christen, Horst Posthaus

**Affiliations:** 1 Institute of Infectious Diseases, Medical Faculty, University of Bern, Bern, Switzerland; 2 Institute of Animal Pathology, Vetsuisse Faculty, University of Bern, Bern, Switzerland; 3 Institut Pasteur, Paris, France; 4 Department of Basic Medical Sciences, Faculty of Medicine and Health Sciences, Ghent University, Ghent, Belgium; Charité-University Medicine Berlin, Germany

## Abstract

*Clostridium perfringens* β-toxin (CPB) is a β-barrel pore-forming toxin and an essential virulence factor of *C. perfringens* type C strains, which cause fatal hemorrhagic enteritis in animals and humans. We have previously shown that CPB is bound to endothelial cells within the intestine of affected pigs and humans, and that CPB is highly toxic to primary porcine endothelial cells (pEC) *in vitro*. The objective of the present study was to investigate the type of cell death induced by CPB in these cells, and to study potential host cell mechanisms involved in this process. CPB rapidly induced lactate dehydrogenase (LDH) release, propidium iodide uptake, ATP depletion, potassium efflux, a marked rise in intracellular calcium [Ca^2+^]_i_, release of high-mobility group protein B1 (HMGB1), and caused ultrastructural changes characteristic of necrotic cell death. Despite a certain level of caspase-3 activation, no appreciable DNA fragmentation was detected. CPB-induced LDH release and propidium iodide uptake were inhibited by necrostatin-1 and the two dissimilar calpain inhibitors PD150606 and calpeptin. Likewise, inhibition of potassium efflux, chelation of intracellular calcium and treatment of pEC with cyclosporin A also significantly inhibited CPB-induced LDH release. Our results demonstrate that rCPB primarily induces necrotic cell death in pEC, and that necrotic cell death is not merely a passive event caused by toxin-induced membrane disruption, but is propagated by host cell-dependent biochemical pathways activated by the rise in intracellular calcium and inhibitable by necrostatin-1, consistent with the emerging concept of programmed necrosis (“necroptosis”).

## Introduction

*Clostridium perfringens* β-toxin (CPB) is a member of the family of heptameric β-pore-forming toxins (β-PFT’s). It is an essential virulence factor of *C. perfringens* type C strains [1], which cause fatal hemorrhagic enteritis in pigs, other animal species, and humans [2]. CPB is closely related to *Staphylococcus aureus* α-toxin, one of the prototype members of β-PFT’s [3]. CPB is secreted as a soluble monomeric protein of ∼35 kDa and causes cell damage by formation of multimeric, ion-selective pores in membranes of susceptible cells [4], [5], [6]. So far, CPB has been shown to be toxic for only a few cell types such as HL-60 cells and endothelial cells, whereas porcine and human fibroblasts, Hela, Vero, CHO, MDCK, Cos-7, P-815, and PC12 cells are insensitive to CPB [4], [5], [7], [8], [9], [10]. We recently demonstrated binding of CPB to intestinal endothelial cells in naturally affected pigs, a human patient, and in experimentally induced lesions in pigs[11], [12], [13]. In addition, CPB was highly toxic to primary porcine and human endothelial cells and induced rapid cell death [9], [10]. It is therefore likely that a direct toxic effect of CPB on endothelial cells in the intestine contributes to the pathogenesis of hemorrhagic and necrotizing enteritis caused by *C. perfringens* type C strains. However, the type of cell death that CPB induces in target cells and the molecular mechanisms involved have not been elucidated yet. β-PFT’s have long been thought to kill target cells simply passively by causing homeostatic disruption and consequent cell lysis upon pore formation [14]. However, several studies have recently shown an active participation of the target cell in the cell death following pore formation and that β-PFT’s influence intracellular signal pathways via non-lytic membrane alterations that can trigger conserved pathways leading to apoptosis or necrosis [14], [15], [16]. Cell death has traditionally been classified according to morphological criteria (apoptosis, autophagic cell death, or necrosis) and whether it is accidental or follows a biochemical program [17], [18]. Although apoptosis has been viewed as the only form of programmed cell death for many years, there is emerging evidence that necrosis can also be executed through distinct and conserved biochemical pathways [19]. This form of cell death has been termed programmed necrosis or “necroptosis”. There is growing evidence that pore-forming toxins produced by bacterial pathogens induce necroptosis in target cells [16], [20], [21], [22]. The objectives of our study were therefore to characterize the type of cell death (apoptosis vs. necrosis) induced by CPB in porcine endothelial cells (pEC), as a likely natural target cell of the toxin, and to differentiate between cell death due to passive events following pore formation (osmotic lysis) and cell death dependent on host cell biochemical pathways. We show here that CPB induces necrotic cell death in pEC, and that cell death, apart from ionic imbalance, depends on the activation of necrostatin-inhibitable, calcium-dependent biochemical events within the host cell, consistent with what is currently being termed necroptosis.

## Materials and Methods

### Cell Culture and Reagents

Aortic pEC were prepared and propagated as described previously [9]. Cells were seeded in DMEM (10% FCS, 1x antibiotic-antimycotic (Gibco), and 20 mM L-glutamine) at a density of 1.33×10^4^ per cm^2^ and grown to confluency for 5 days. Only cells from passages 3–8 were used. To suppress K^+^ efflux, high potassium medium (5 mM NaCl, 140 mM KCl, 10 mM Hepes, 1.3 mM CaCl_2_, 0.5 mM MgCl_2,_ 0.36 mM K_2_HPO_4,_ 0.44 mM KH_2_PO_4_, 5,5 mM D-glucose, 4.2 mM NaHCO_3_) was used instead of regular Hank’s balanced salt solution (HBSS), as described previously [23]. Recombinant CPB (rCPB) was expressed as an inactive Nus-Tag™ fusion protein (Novagen) and subsequently purified after enzymatic cleavage as described previously [9]. Toxin concentrations used in our experiments were chosen based on previous results [9] with a low toxin dose of 30 ng/ml (equivalent to 0.86 nM) and a high toxin dose of 250 ng/ml (equivalent to 7.17 nM). Toxin pre-incubated with a neutralizing antibody (mouse monoclonal anti-CPB 10A2 [mAb-CPB], Center for Veterinary Biologics, USDA) served as a control. Staurosporine was from Sigma, Necrostatin-1 (Nec-1) from Biomol (Enzo), BAPTA-AM, the non-peptide calpain inhibitor PD150606, calpeptin (Z-Leu-Nle-CHO), Q-VD-OPh (QVD), and zVAD-fmk from Calbiochem. Recombinant polyhistidine-tagged and GFP-labeled annexin V was expressed in *E. coli* and purified as described previously [24].

### PI/annexin V FACS Analysis

Cells were grown in 6-well plates and exposed to rCPB or staurosporine for different periods of time. Cells were then labeled directly in the well with 10 μg/ml propidium iodide (PI, Sigma) and 4.5 μg/ml GFP-annexin V for 15 min at room temperature. Floating cells were collected in phenol red-free DMEM and adherent cells gently detached by mild trypsinization [25]. The two fractions were pooled and centrifuged for 5 min at 700×g, resuspended in 300 μl phenol red-free DMEM and analyzed by FACS on a BD FACScan (Becton Dickinson). For each analysis, 20′000 gated cells were counted. The total cell number was determined using an automated cell counter (Invitrogen Countess).

### LDH Release Assays

Lactate dehydrogenase (LDH) activity in cell culture supernatants was determined by an enzyme assay (Roche Cytotoxicity Detection Kit^PLUS^) according to the manufacturer’s instructions. Control cell lysates were prepared with 0.1% Triton X-100 ( = 100% cell death).

### Determination of Intracellular ATP

pEC grown in 96 well plates were exposed to rCPB for different periods of time, rinsed with PBS, shock frozen on the plate with liquid nitrogen and stored at −80°C. ATP was extracted by incubating cells with 100 µl 4% perchloric acid for 20 min on ice. The pH was adjusted to 7.7 by adding 10 µl of 15% potassium hydroxide. ATP levels were determined using the ENLITEN Kit (Promega) and an EnSpire Multilabel Reader (Perkin Elmer). ATP levels were calculated from an ATP standard curve and were adjusted to protein concentration determined by the Bradford method (Pierce).

### DNA Fragmentation

Cells grown in 25 cm^2^ cell culture flasks were incubated with either rCPB, H_2_O_2_, or staurosporine, and internucleosomal DNA was isolated with the Genzyme TACS Apoptotic DNA-Laddering Kit (Trevigen) according to the manufacturer`s guidelines. Isolated DNA was then run on a 1.5% TreviGel (Genzyme) stained with ethidium bromide and laddering documented using a Syngene U: Genius Gel Documentation Systeme (Syngene).

### Western Blot Analyses

Cells grown in 25 cm^2^ cell culture flasks were exposed to rCPB or staurosporine for different periods of time, washed with PBS and lysed in 1 ml RIPA-buffer (50 mM Tris-HCl, pH 7.4, 150 mM NaCl, 1% NP-40, 0.25% Na deoxycholate, 1 mM EDTA and 0.1% SDS). Lysates were centrifuged at 14′000 g for 10 min. Protein measurements were performed using the BCA method (Pierce). Lysates were boiled in SDS sample buffer (Laemmli buffer) and equal amounts of protein separated on 12% SDS-polyacrylamide gels. For the determination of HMGB-1 release, cells were incubated in a minimum amount of medium. Cell supernatant was harvested at indicated time points and boiled in SDS sample buffer. After electrophoresis of samples, proteins were transferred to nitrocellulose membranes and probed with polyclonal cleaved caspase-3 specific antibodies (Cell Signaling, 1∶1000) or monoclonal anti-HMGB-1 antibodies (Cell Signaling, 1∶1000). Immunoreactive bands were detected using the Odyssee Infrared Imager System (Li-Cor) as described previously [9].

### Transmission Electron Microscopy (TEM)

Cells grown on culture dishes were exposed to rCPB for 6 or 12 h, fixed in 4% formaldehyde in a 0.121 M sodium cacodylate buffer for 24 h and post-fixed for 12 h with osmium tetroxide. The cells were then embedded in an epoxy resin (LX-112 Ladd Industries) and sections were prepared with a Reichert Jung Ultracut E ultra-microtome. Semithin sections (2 µm) were stained with toluidine blue to select the most appropriate regions for ultrathin sectioning. Ultrathin sections (90 nm) were made and stained with uranyl acetate and lead citrate before examining under a Jeol EX II transmission electron microscope at 80 kV.

### Light Microscopy and Immunofluorescence

Confluent pEC grown in LabTek slides (Nunc) were incubated with medium containing rCPB or staurosporine. Light microscopy, immunofluorescence staining and detection was performed as previously described [9]. Fixed and permeabilized cells were stained using monoclonal anti-HMGB-1 antibodies (Cell Signaling, 1∶100) and goat-anti-mouse Alexa Fluor 594 labeled secondary antibodies (Molecular Probes). Nuclei were stained with Hoechst 33258 (Molecular Probes).

### Determination of Intracellular K^+^

Cells were grown in 6-well plates and exposed to rCPB or toxin pre-incubated with mAb-CPB for different periods of times, washed 3 times with 25 mM MOPS (pH 7.0) containing 150 mM glucose, and lysed in 150 ul 1% Triton X-100 in PBS. 45 µl of lysate was incubated with CD222 (1 µM, Molecular Probes) for 15 min at RT and fluorescence was measured on a Fluoroskan II using an excitation wavelength of 380 nm and an emission wavelength of 475 nm (Knapp, Maier et al. 2010). Results are expressed as a percentage of the signal in untreated cells ( = 100%) and cells lysed with 0.2% Triton X-100 for 5 min at 37°C ( = 0%).

### Determination of Intracellular Ca^2+^

Intracellular calcium was determined with the calcium sensitive dye Fluo-4 Direct™ (Fluo-4 Direct™ Calcium Assay Kit, Invitrogen) according to the manufacturer’s instructions. Briefly, an equal volume of 2X Fluo-4 Direct™ was added directly to confluent pEC grown in a black 96-well plate. Cells were loaded with the fluorescent dye protected from light for 30 min at 37°C with an additional 30 min at RT. Extracellular Ca^2+^was adjusted to a final concentration of 3 mM. After exposure to rCPB, fluorescence was measured by bottom reading every minute for 4 h at 37°C using a Varioskan Flash (Thermo Scientific) at an excitation wavelength of 494 nm and an emission wavelength of 516 nm. Data are expressed as a ratio of the untreated control relative to the starting ratio using the following equation: ΔF/F = ((T_n_/C_n_) – (T_0_/T_0_))/(T_0_/C_0_), where F = fold change in fluorescence, T = average of the readings of the toxin-treated sample replicates, C = average of the readings of the control sample replicates, n is the time point post toxin addition and 0 is the initial reading [16].

### Statistical Analysis

Data are presented as mean +/− SD. Multiple comparisons were performed by 1-way analysis of variance (ANOVA) using Dunnett’s post-hoc analysis to compare against control, and Tukey post-hoc analysis to analyze the effect of inhibitors. Kruskal-Wallis with Dunn’s post-hoc test was used to analyze non-Gaussian distributed data. A P-value of <0.05 was considered to be statistically significant.

## Results

### Morphological Changes and Kinetics of CPB-induced Cell Death

**Figure 1:**
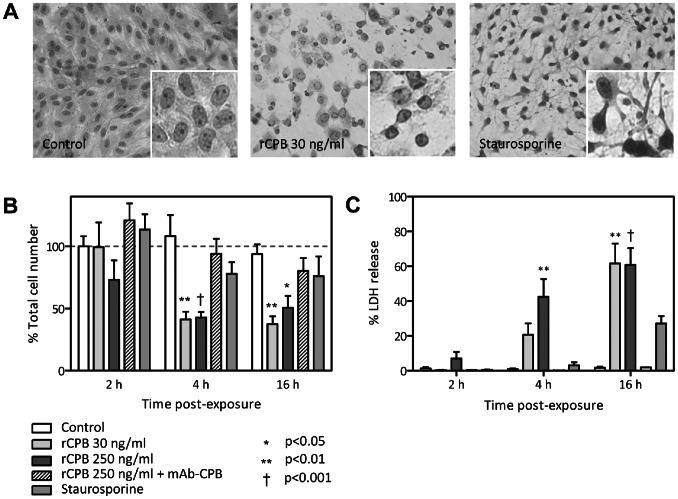
Morphological changes and LDH release induced by rCPB in pEC. (A) pEC were either left untreated (white) or exposed to rCPB or staurosporine (200 nM) for 4 h. Cell morphology was visualized using phase contrast microscopy. (B) Changes in total cell number (determined using automated cell counter) of pEC after 2, 4 and 16 h incubation with control medium, rCPB at indicated concentrations, neutralized rCPB using monoclonal anti-CPB antibodies, or staurosporine. Bar graphs represent the mean ± SEM of n = 3–6 independent experiments. Statistical difference to control cells was assessed by 1-way ANOVA and Dunnnet post-hoc test. *P<0.05, **P<0.01, †P<0.001. (C) The supernatants of pEC cultures from B were analyzed for LDH activity to determine LDH release after different times of exposure. Bar graph shows summary of results from 3 to 6 independent experiments expressed as percentage of activity compared to lysed control cells ( = 100%). Statistical difference to non-treated control cells was assessed by 1-way ANOVA and Dunnnet post-hoc test. *P<0.05, **P<0.01, †P<0.001.

To study the type of cell death induced by CPB, confluent monolayers of pEC were incubated with either a low (30 ng/ml equivalent to 0.86 nM) or high dose (250 ng/ml equivalent to 7.17 nM) of rCPB. Staurosporine served as positive control for apoptosis [26]. In line with our previous observation [9], the morphology of rCPB-treated cells started to change markedly already after 1 h. At both toxin concentrations, cell borders became retracted and cells subsequently rounded up, while nuclei appeared to remain intact (Fig. 1A, 4 h after exposure). In contrast, staurosporine induced cytoplasmic shrinking and frequent nuclear condensation, while cells remained attached. CPB caused a marked reduction in the cell number at 4 and 16 h after exposure, which was mirrored by a corresponding increase in LDH release (Fig.1B,C). In contrast, staurosporine treatment did not cause a significant reduction in the cell number, or a significant increase in LDH release; neither did pEC exposed to rCPB pre-incubated with neutralizing anti-CPB antibodies (Fig. 1B,C). These results indicate that rCPB causes progressive plasma membrane disruption and lysis of pEC.

**Figure 4:**
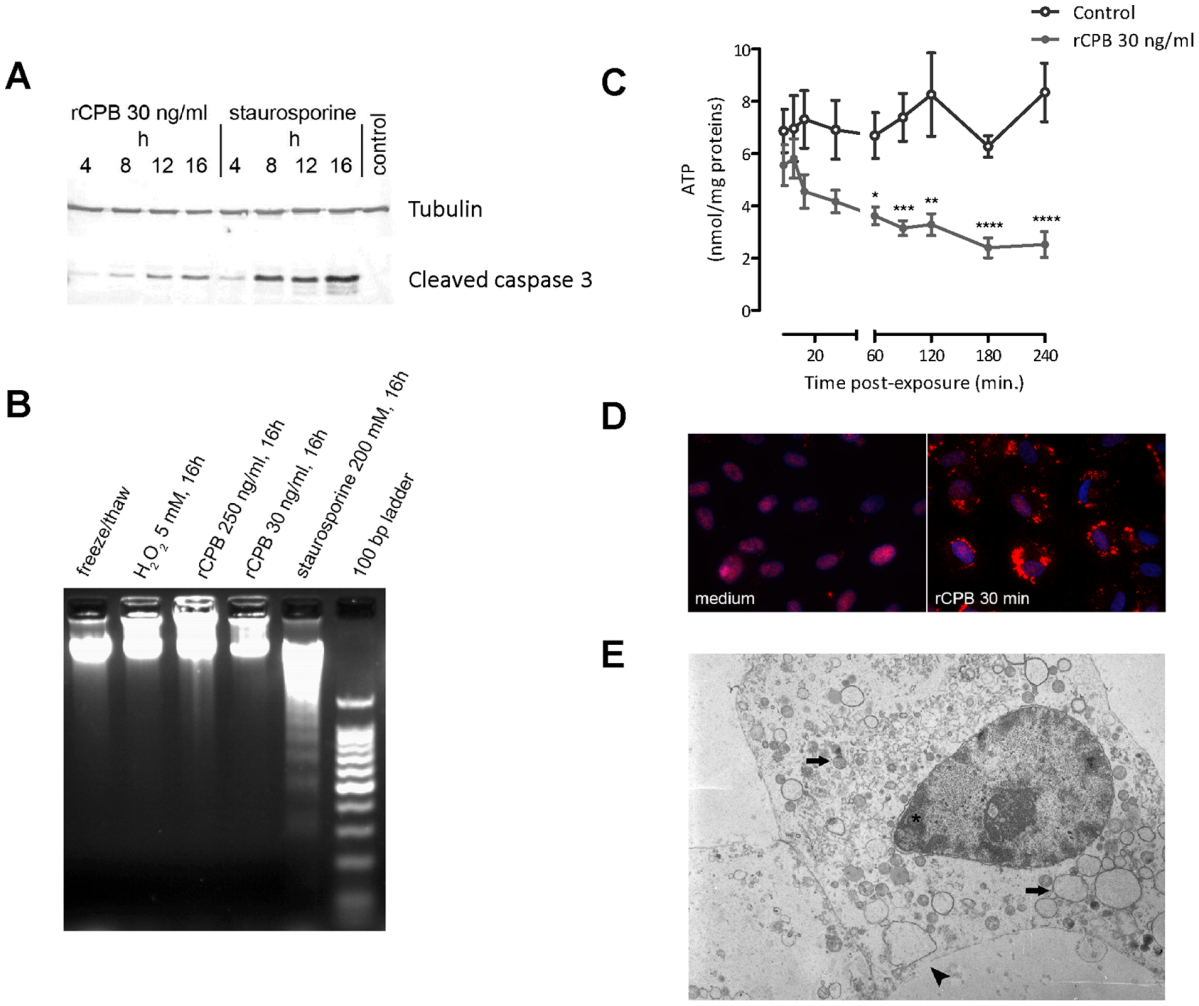
pEC exposed to rCPB do not exhibit apoptotic but typical features of necrotic cell death. (A) Presence of cleaved caspase 3 was assessed by Western blotting at different time points after exposure to 30 ng/ml rCPB or staurosporine. Tubulin was used as loading control. Representative results of 3 independent experiments. (B) Internucleosomal DNA fragmentation was assessed after 16 h of exposure to rCPB. Staurosporine was used as a control for apoptosis, while incubation with 5 mM of H_2_O_2_ or freeze thawing served as controls for necrosis. Representative results of 3 independent experiments. (C) Intracellular ATP levels were determined at the indicated time points. Results represent mean ± SEM of 3 independent experiments. Statistical difference to untreated control cells was assessed by 2-way ANOVA and Bonferroni multiple comparisons test. *P<0.05, **P<0.01, †P<0.001, ††P<0.0001. (D) Cytoplasmic translocation of HMGB-1 (red) was detectable by immunofluorescence in pEC already 30 min after exposure to 30 ng/ml rCPB. Control cells incubated with toxin-free medium exhibit typical nuclear localization. Nuclear counterstain with Hoechst 33258 (blue). (E) Electron microscopic image of pEC 6 h after incubation with 30 ng/ml rCPB. Cells exhibit small irregular clumps of chromatin abutting to the nuclear membrane (asterisk), swelling of cell organelles with disappearance of the elongated mitochondria with cristae (arrows), and plasma membrane discontinuities (arrowhead).

### Characterization of CPB-induced Cell Death by FACS Analysis

**Figure 2:**
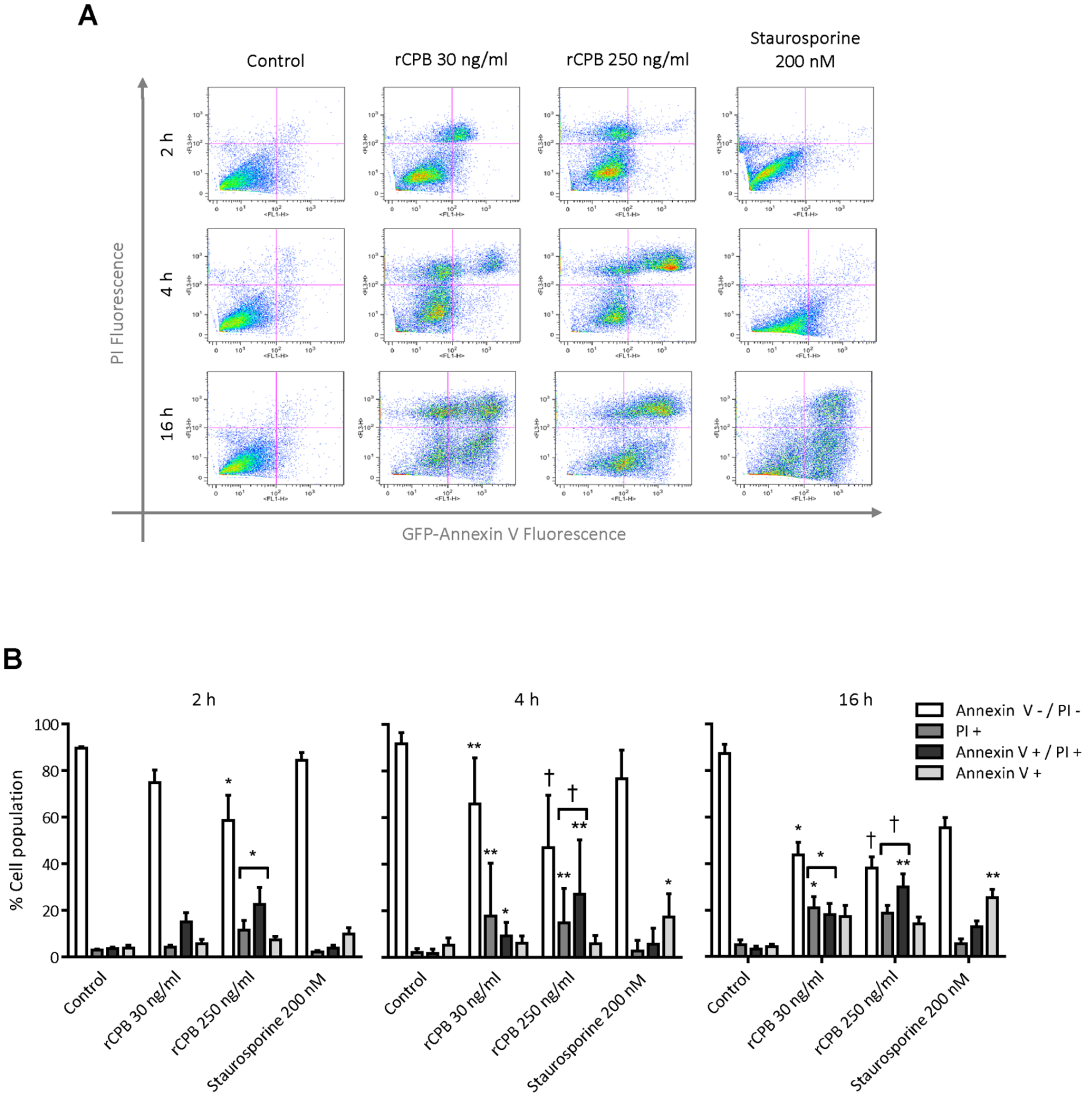
Characterization of rCBP-induced cell death by FACS analysis. pEC were either left untreated or exposed to 30 or 250 ng/ml of rCPB, or staurosporine, detached from the culture dishes by gentle trypsinization, and analyzed by FACS for PI uptake and GFP-annexin V staining at 2, 4 and 16 h of incubation. (A) Representative cytograms and bar graphs (B) in which cells in the lower left quadrant (PI/annexin V double-negative) were considered to be viable (white), cells in the upper left quadrant (PI single-positive) to be necrotic (gray), cells in the lower right quadrant (annexin V single-positive) to be apoptotic (light gray), and cells in the upper right quadrant (PI/annexin double-positive) to be either necrotic or secondary necrotic after apoptosis (dark gray). Bar graphs show quantitative summary of FACS analysis from 3 to 6 independent experiments. Results are expressed as mean ± SD. Statistical difference to control cells was assessed by non-parametric Kruskal-Wallis and Dunn’s post-hoc test. *P<0.05, **P<0.01, †P<0.001.

We further characterized the type of cell death induced by rCPB using PI/annexin V staining and FACS analysis of pEC that were not lysed yet by the toxin. During apoptosis, phosphatidylserine at the inner leaflet of the plasma membrane is flipped to the outside of the cell, which can be detected by the binding of annexin V to intact cells. PI is charged and can only be taken up by cells that have a compromised membrane. At 2 and 4 h after exposure to rCPB, a significant proportion of cells were already PI positive (either single or double-positive) compared to control cells, with a larger increase and shift to PI/annexin V double-positive cells at the higher dose (Fig.2 A,B). At 16 h after exposure, the fraction of viable cells has further decreased, and the number of PI positive cells (single and double-positive cells combined) was also significantly increased at the low dose of toxin. A significant increase in annexin V single-positive cells could not be observed at any of these time points, whereas a significant increase in annexin V positive cells was already observed at 4 h after treatment with staurosporine, which was further increased at 16 h post-treatment. At this time point, some staurosporine-treated cells have started to become PI/annexin V double-positive, indicative of secondary necrosis.

### Uptake of PI and LDH Release is not Significantly Inhibited by the Caspase Inhibitor QVD, but the Necroptosis Inhibitor Necrostatin-1

**Figure 3:**
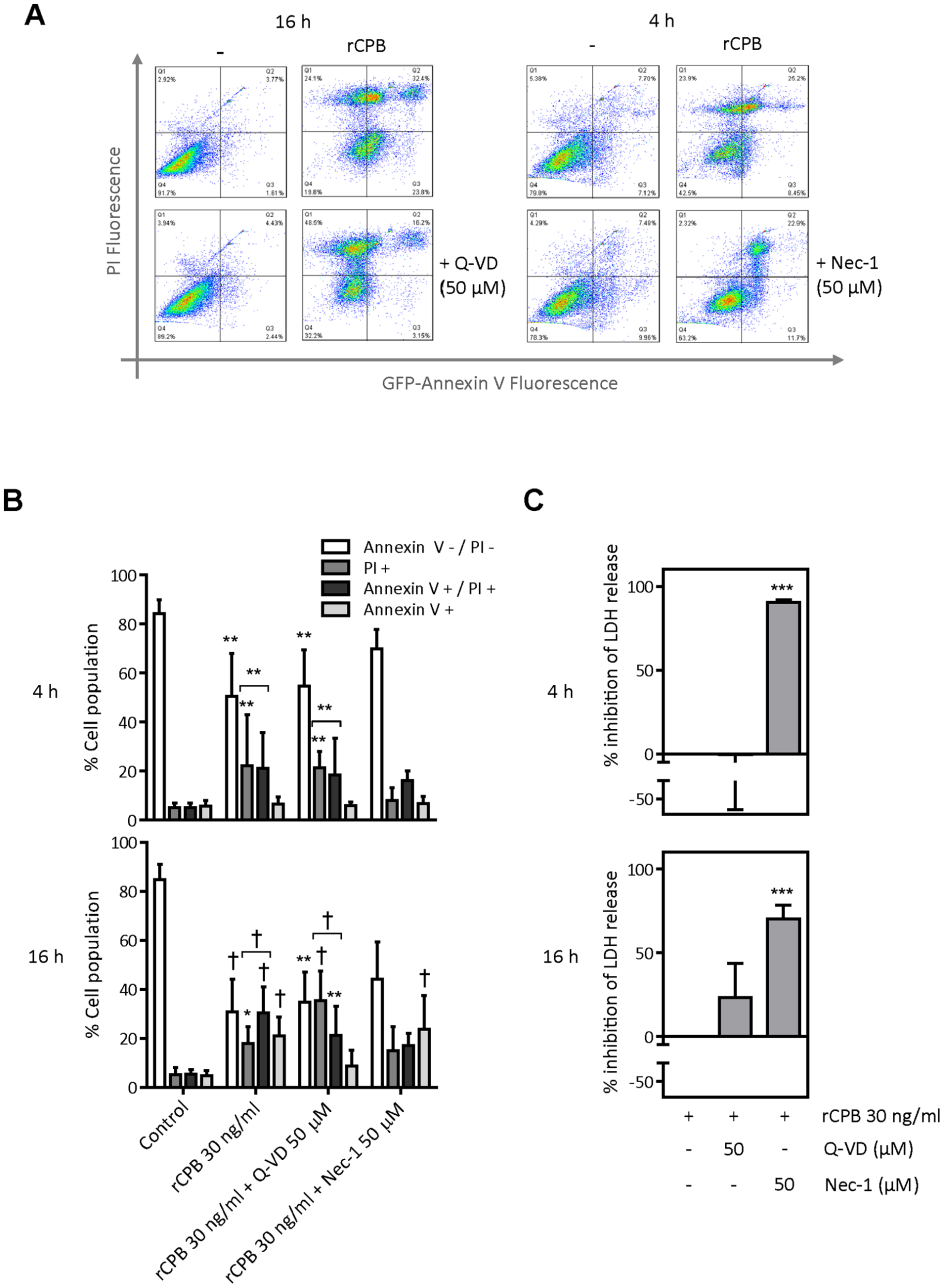
Effect of QVD and nectrostatin-1 on CPB-induced cell death. (A) Representative cytograms of cells exposed to 30 ng/ml of rCPB in the absence or presence of QVD (50 µM) (at 16 h) or necrostatin-1 (Nec-1, 50 µM) (at 4 h) (B) Bar graphs show quantitative summary of FACS analysis from 4 to 6 independent experiments. Results are expressed as mean ± SD. Same key as in Figure 2B. Statistical difference to control cells was assessed by non-parametric Kruskal-Wallis and Dunn’s post-hoc test. *P<0.05, **P<0.01, †P<0.001. (C) Bar graphs show % inhibition of LDH release of cells treated with QVD or Nec-1 compared to cells treated with toxin only (control) and represent results from 3 to 5 independent experiments. Statistical difference to control was assessed by 1-way ANOVA and Dunnnet post-hoc test, †P<0.001.

Because the PI/annexin V double-positive cells in rCPB-treated cultures could be derived from secondary necrosis following apoptosis (although no significant increase in annexin V single-positive cells was observed in the time-course experiment), cells were co-incubated with the broad-spectrum caspase inhibitor QVD [27]. Co-treatment with QVD significantly inhibited the staurosporine-induced increase in annexin V single- and double-positive cells (not shown). While QVD had no effect on the staining pattern at 4 h, QVD treatment appears to have caused a shift from PI/annexin V double-positive to PI single-positive cells at 16 h after toxin exposure (Fig. 3A,B). Consistent with rCPB not causing LDH release primarily due to secondary necrosis, QVD treatment had no effect on LDH release at 4 h and only slightly, but non-significantly, inhibited LDH release at 16 h post-exposure (Fig. 3C). Treatment with zVAD (50 µM) also did not significantly reduce LDH release induced by rCPB (data not shown). Different to the experiments shown in Figure 2, toxin exposure was associated with a small increase in annexin V single-positive cells (compared to PI single and double-positive cells), which was inhibited by QVD, suggesting that some apoptosis, at least its activation, may occur.

In contrast to QVD, co-incubation with the necroptosis inhibitor Nec-1 [28] almost completely prevented the increase in PI staining, whether single- or double-positive (Fig. 3A,B). This was mirrored by an almost complete inhibition of LDH release at 4 and 16 h after toxin exposure (Fig. 3C). These results indicate that CPB-intoxicated pEC do not primarily die by apoptosis, and that necrotic cell death is dependent on a Nec-1-inhibitable cellular program.

### CPB-treated pEC Exhibit Some Caspase 3 Activation, but Primarily Die by Necrosis

To further confirm that apoptosis is not the major pathway by which CPB-intoxicated pEC die, we assessed two further hallmarks of apoptotic cell death, caspase 3 activation and internucleosomal DNA fragmentation. Although some caspase 3 cleavage (i.e., activation) was detected 12 h after exposure to rCPB (Fig. 4A), consistent with the small increase in annexin V single-positive cells at 16 h in Fig. 3B, staurosporine-treated cells exhibited much higher levels of activated caspase 3. More importantly, in contrast to staurosporine, no appreciable DNA fragmentation was detected 16 h after incubation with rCPB (Fig. 4B).

To verify that intoxication with rCPB primarily results in necrotic cell death of pEC, we assessed further parameters characteristic of necrotic cell death. In line with CPB inducing necrotic cell death, exposure of pEC to the toxin resulted in rapid depletion of intracellular ATP levels (Fig. 4C). Moreover, rCPB induced translocation of HMGB-1 from the nucleus to the cytoplasm and later into the supernatant of pEC (Fig. 4D). Cytoplasmic translocation of HMGB-1 was already visible after 30 min and HMGB-1 could be detected in cell culture media by western blotting after 6 h (data not shown). At 6 h after exposure to rCPB, pEC showed typical ultrastructural features of necrosis, such as chromatin condensation, swelling of intracellular organelles, and plasma membrane disruption (Fig. 4E). At 12 h, these changes were even more pronounced and plasma membranes were markedly disrupted (data not shown).

### Intoxication of pEC with CPB is Associated with an Increasing Efflux of Potassium and Rise in Intracellular Calcium

**Figure 5:**
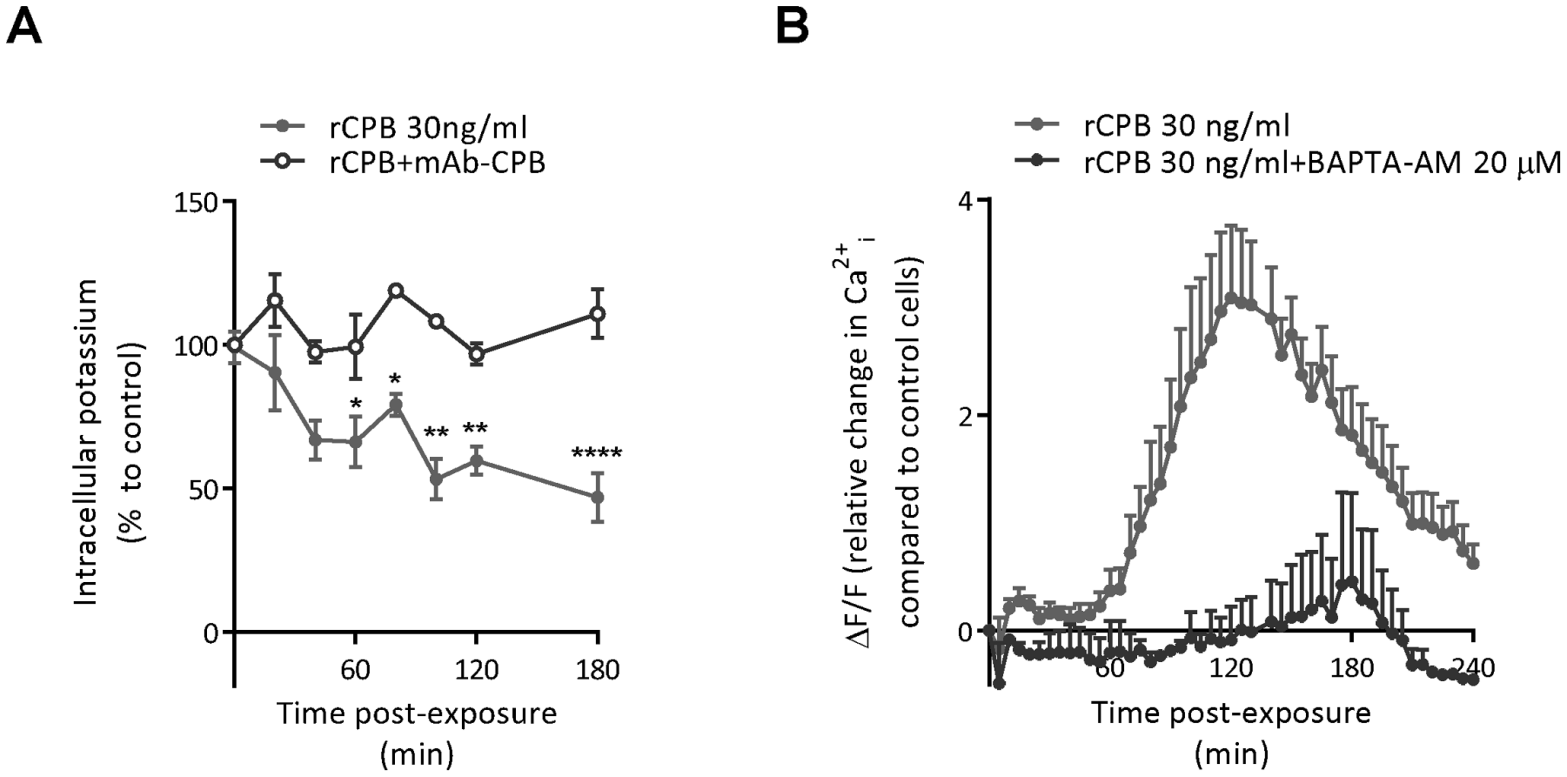
rCPB-induced efflux of K^+^ and accumulation of intracellular Ca^2+^. (A) Incubation of confluent pEC with 30 ng/ml rCPB resulted in rapid and significant reduction of intracellular K^+^. rCPB preincubated with mAb-CPB did not cause K^+^ efflux. Values represent means ± SEM from 3 independent experiments. Statistical difference to cells treated with neutralized toxin ( = control) was assessed by 2-way ANOVA and Bonferroni multiple comparisons test. *P<0.05, **P<0.01, ****P<0.0001. (B) Intracellular calcium ([Ca^2+^]_i_) levels were measured by Fluo-4 fluorescence in the presence of 3 mM extracellular CaCl_2_. Results are expressed as mean ± SEM from 3 independent experiments.

Having established that rCPB primarily causes necrotic cell death in pEC, we evaluated whether CPB also induced K^+^ efflux and changes in intracellular calcium homeostasis, two events typically observed after plasma membrane pore formation by β-PFT’s [29]. Exposure of pEC to 30 ng/ml of rCPB led to a rapid and sustained reduction of intracellular K^+^ (Fig. 5A). This occurred before significant cell death was observed (*cf.* *Fig. 1B&C*). Preincubation of the toxin with a neutralizing antibody completely inhibited K^+^ efflux, consistent with K^+^ efflux being the result of ion-selective pore formation [4], [5]. rCPB also induced a rapid, small increase in [Ca^2+^]_i_ that was followed by a secondary more sustained and more pronounced increase in [Ca^2+^]_i_ at 60 min after exposure (Fig. 5B). The intracellular calcium chelator BAPTA-AM (20 μM) inhibited both the small initial increase and the more pronounced secondary increase, suggesting an intracellular origin of the calcium signal (Fig. 5B). Exposure to 250 ng/ml of rCPB led to a more rapid and more pronounced increase in [Ca^2+^]_i_ (data not shown). Noteworthy, the secondary increase occurred at a time when ATP levels had already dropped to 50% below baseline (*cf.* *Fig. 4C*), suggesting that the increase in [Ca^2+^]_i_ may not entirely be the result of influx of extracellular calcium through the plasma membrane pores formed by the toxin.

### High Extracellular K^+^, Prevention of the Intracellular Rise in Ca+, and Inhibition of Calpain Activity All Inhibit CPB-induced LDH Release

**Figure 6:**
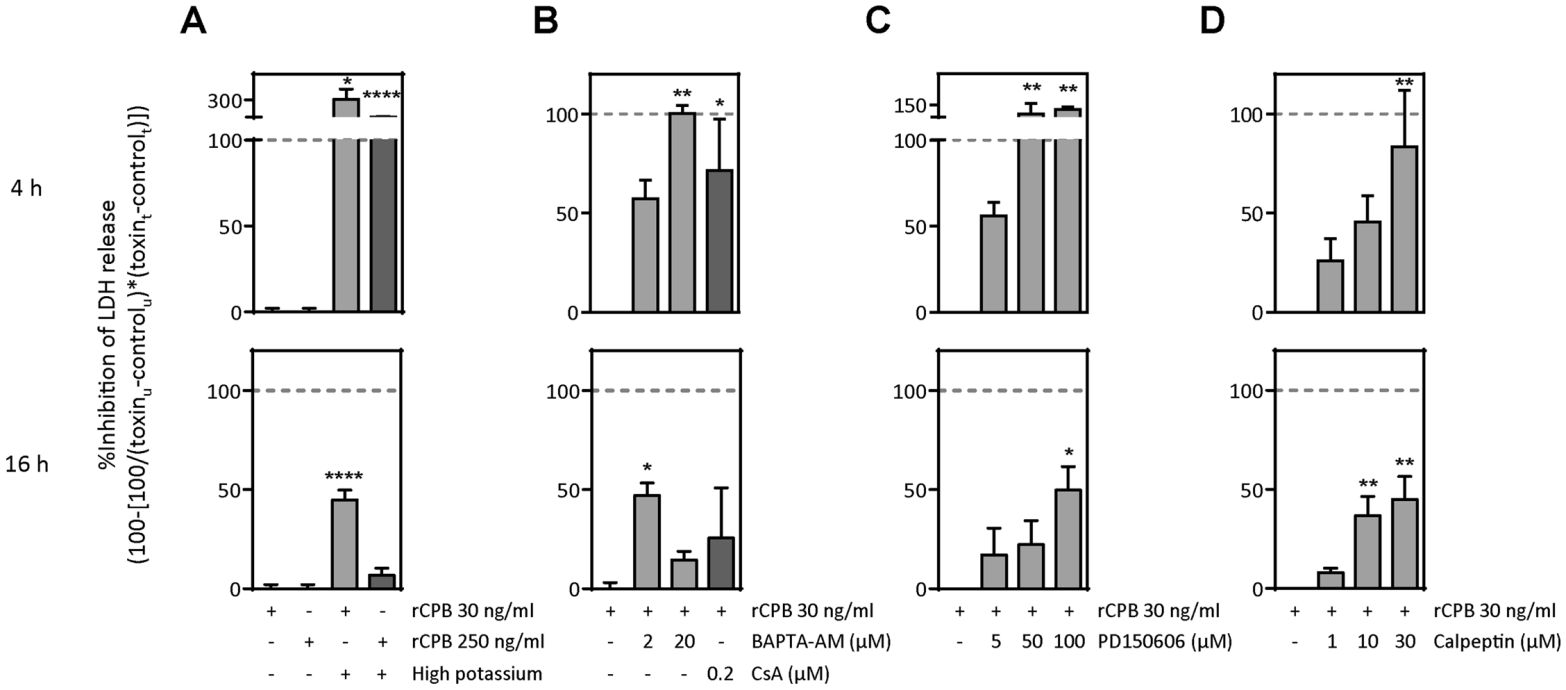
Attenuation of rCPB-induced necrosis by high extracellular K^+^, chelation of intracellular Ca^2+^, and calpain inhibition. (A) Suppression of potassium efflux by high K^+^ medium (140 mM) significantly inhibited rCPB-induced LDH release from pEC at 4 h, and still reduced LDH release at 16 h, when cells were exposed to 30 ng/ml rCPB. (B) Chelation of intracellular Ca^2+^ with BAPTA-AM (2 or 20 µM) or treatment with cyclosporin A (200 nM) markedly inhibited rCPB induced LDH release at 4 h, and to a lesser extend at 16 h post-exposure. LDH release of rCPB-treated cells in absence or presence of increasing concentrations of PD150606 (C) or calpeptin (D) was measured at 4 and 16 h. Bar graphs show summary of results from 2 to 5 independent experiments, which are expressed as percentage ± SEM relative to rCPB-treated cells in the absence of inhibitor or presence of low K^+^ medium ( = control). Statistical difference to control was assessed by 1-way ANOVA and Dunnnet post-hoc test. *P<0.05, **P<0.01. u = untreated cells, t = treated cells.

To determine the importance of K^+^ efflux in rCPB-induced necrosis, pEC were incubated in medium which contained high levels of K^+^ (∼140 mM). Under these conditions (which suppress passive diffusion of intracellular K^+^ through the pores, [23]), LDH release was completely inhibited at 4 h post-exposure to either high or low toxin, and still partially inhibited at 16 h in the case of low toxin (Fig. 6A). The effect of extracellular Ca^2+^ depletion on rCPB-induced cell death could not be studied, as cells incubated in HBSS without Ca^2+^ did not survive long enough to carry out this experiment. However, to address the question whether the rise in intracellular Ca^2+^ is directly involved in rCPB-induced cell death, cells were pre-incubated with two different concentrations of BAPTA-AM (Fig. 6B). Chelation of intracellular Ca^2+^ (*cf.* *Fig. 5B*) was associated with dose-dependent inhibition of LDH release, which was more pronounced at 4 than at 16 h post-exposure. At 20 µM, BAPTA-AM treatment became progressively toxic to the cells (hence the lack of effect at 16 h post-exposure). Partial protection from rCPB-induced cell death was also observed when cells were preincubated with cyclosporin A (CsA) (Fig. 6B), which binds to the cyclophilin family of proteins, thereby preventing the activation of calcineurin (a calcium-dependent protein phosphatase) or opening of the mitochondrial permeability transition pore, suggesting that activation of calcium-dependent, host-cell biochemical pathways are involved in rCPB-induced cell death.

Calpains, a family of calcium-activated proteases that cause cytoskeletal and lysosomal disruption [27], have previously been shown to be involved in cell death induced by *C. septicum* α-toxin [16]. To address if activation of calpains is also involved in CPB-induced cell death, cells were co-treated with either PD150606 (a non-competitive calpain inhibitor specifically directed towards the calcium-binding site of the protein) or calpeptin (a peptide inhibitor that blocks calpain activity irreversibly). Consistent with rCPB-induced cell death not being simply a passive event, both inhibitors dose-dependently inhibited LDH release (Fig. 6C&D). CPB-induced release of LDH was completely inhibited by PD150606 and calpeptin at 4 h after exposure and was reduced by approximately 50% at 16 h of exposure, indicating that activation of host cell calpains plays an important role in rCPB-induced necrosis.

## Discussion

Although CPB has been shown to form multimeric, ion-selective pores in lipid bilayers and human promyelocytic leukemia (HL-60) cells [4], [5], and to rapidly induce cell death in primary endothelial cells [8], [9], [10], little attention has been given to the host cell response mechanisms induced by CPB pore formation. We recently postulated that endothelial cells are primary natural target cells for CPB [9] and thus investigated the type of cell death that CPB causes in pEC more closely. Our results demonstrate that rCPB rapidly induces necrosis in these cells. Although the FACS analyses and the evaluation of caspase 3 activation showed some evidence for activation of apoptosis, pEC exposed to rCPB exhibited the typical morphological and biochemical features of cells that had died by necrosis: progressive plasma membrane disruption, detectable ultrastructurally and by LDH release/PI uptake, loss of cellular ATP, chromatin condensation, and translocation of HMGB-1 from the nucleus to the cytoplasm followed by release into the cell culture medium. Caspase inhibition had no significant effect on rCPB-induced LDH release and PI uptake. Caspase inhibition has previously been shown to sensitize certain cell types to tumor necrosis factor alpha (TNFα)-induced necrosis [30]. Intriguingly, FACS analyses of pEC exposed to rCPB in the presence of QVD showed a slight shift towards PI/annexin V double staining, suggesting that caspase inhibition may also sensitize cells to necrosis induced by pore-forming toxins, which do not appear to act via death domain receptors.

There is increasing evidence that pore formation by β-PFT’s can activate intracellular signaling pathways leading to different cellular reactions such as apoptotic or necrotic cell death, cell survival or inflammatory reactions [15]. We show here that Nec-1 almost completely inhibits LDH release and PI uptake in pEC intoxicated with rCPB, indicating that activation of host cell-dependent mechanisms are involved in the necrotic cell death induced by CPB. Nec-1 has initially been identified in a small-molecule library screen to potently inhibit TNFα-induced necrotic cell death (“necroptosis”) of the human monocytic cell line U937 in the presence of zVAD-fmk or murine L929 cells [28]. Later on, the death domain receptor-associated adaptor kinase RIP1 was identified as a specific cellular target of Nec-1 in FADD-deficient Jurkat T cells [31]. Phosphorylation-driven assembly of a RIP1-RIP3 complex has been shown to regulate TNFα-induced programmed necrosis in FADD-deficient Jurkat T cells or zVAD-fmk-pretereated L929 cells, or necroptosis induced by vaccinia virus infection [32]. While RIP1 has clearly been shown to be essential for TNFα-induced necroptosis, recent evidence suggests that the inhibitory effect of Nec-1 on necroptosis can also be explained by its action on other targets than RIP1 [33]. While we could detect RIP1 and RIP3 in pEC cell lysates, we were unable to demonstrate RIP1-RIP3 complex formation in toxin-treated cells by co-immunoprecipitation (Autheman et al., unpublished). Whether this was due to the complex not being formed or the inability to detect complex formation in the porcine cells is currently unclear. Thus, further experiments are required to identify the molecular target of Nec-1 in our system. Nevertheless, the inhibitory effect of Nec-1 on LDH release and PI uptake clearly indicate that necrotic cell death induced by CPB is not merely a passive event.

Recent studies on aerolysin, listeriolysin and *C. speticum* α-toxin have shown that intracellular potassium and calcium are central regulators of the cellular response to PFT’s [16], [23], [29]. The rapid K^+^ efflux observed in our experiments is an immediate consequence of plasma membrane pore formation by rCPB. This was followed by a pronounced rise in intracellular Ca^2+^. Inhibition of both of these events reduced LDH release, indicating these two events are important triggers for the activation of intracellular cascades that result in necrosis of pEC, following pore formation by CPB. Inhibition of LDH release by cyclosporin A suggests that mitochondrial dysfunction may play an important role in CPB-induced necrosis. This may explain the rapid loss in ATP generation upon intoxication.

In further support of this notion are our results with the calpain activity inhibitors. Calpains are Ca^2+^-dependent proteases that cleave multiple cellular targets resulting in cytoskeletal rearrangements, lysosomal rupture, and further increase of intracellular Ca^2+^ [27]. A common feature of intoxication with pore-forming toxins is a rapid rise of intracellular Ca^2+^ [16], [34]. This has also been shown to be the case for HL-60 cells exposed to CPB [4]. In line with this observation, exposure of pEC to rCPB led to a rapid and massive increase in intracellular Ca^2+^. Using two chemically distinct calpain inhibitors, we could show that necrosis of pEC induced by CPB was not merely a passive event but dependent on biochemical, host cell-driven programs involving activation of calpains, consistent with the cell death being necroptosis. Such an involvement of Ca^2+^-dependent calpain activation has previsouly been shown for the induction of necroptotic cell death in murine myoblast cells by the clostridial β-PFT *C. septicum* α-toxin [16]. It is likely that calpain activation occurs as a result of the large and sustained increase in intracellular Ca^2+^, as scavenging of intracellular Ca^2+^ with BAPTA-AM markedly inhibited rCPB-induced LDH release. The inhibitory effect with CsA may indicate that not only pore formation but also mitochondrial dysfunction contributes to the marked increase in intracellular calcium. There is growing evidence that bacterial β-PFT’s such as *Staphylococcus aureus* α-toxin, *Escherichia coli* hemolysin, *Helicobacter pylori* VacA, *C. perfringens* ε-toxin and *C. perfringens* enterotoxin also induce cell death by necroptosis [6], [21], [22], [35]. Our results strongly suggest that CPB can be added to the growing number of bacterial pore-forming toxins that cause tissue damage by inducing necroptosis in their target cells. It remains to be shown whether these toxins may ultimately act via a common pathway, which could involve activation of a necrostatin-inhibitable target, such as RIP1 [36].

Induction of necrosis via evolutionally conserved intracellular pathways by CPB could indeed be of biological importance in *C. perfringens* infections. CPB is the major virulence factor of *C. perfringens* type C strains, which cause a severe necrotizing and hemorrhagic enteritis in affected hosts, such as pigs or humans [2]. Induction of necrosis in endothelial cells, as shown here, correlates to the timely development of lesions observed during experimental and natural *C. perfringens* type C infection in pigs. Early lesions in experimental infections were focal but rapidly progressing capillary dilation in small intestinal villi, followed by hemorrhage [13]. Later stage lesions observed in naturally affected pigs are vascular necrosis and thrombosis and a marked infiltration of neutrophils into affected areas [11], [37]. Vascular endothelial cell necrosis induced by CPB could thus initiate local vascular damage and the release of pro-inflammatory cell components, such as HMGB-1, from endothelial cells. This could therefore induce local hemorrhage, hypoxic tissue necrosis, and further tissue damage by an inflammatory reaction. As an anaerobic pathogen, *C. perfringens* type C would benefit from such hypoxic tissue damage leading to massive proliferation at the site of initial damage, further toxin secretion and rapid progression of the disease resulting in the typical segmental necrosis of the small intestine.

### Conclusions

The results of this study show that the β-PFT CPB, the main virulence factor of *C. perfringens* type C strains, primarily causes necrosis in primary porcine endothelial cells. Necrosis induced by CPB includes the activation of host cell-driven biochemical events involving calpain activity, consistent with the cell death being necroptosis. Induction of necroptosis via pore formation in endothelial cells by CPB could play a major role in the development of lesions observed in *C. perfringens* type C enteritis. Early cytoskeletal and morphological changes in intestinal endothelial cells could explain the massive hemorrhage observed in naturally and experimentally induced gut lesions, whereas release of pro-inflammatory intracellular substances, such as HMGB-1, from endothelial cells could contribute to the massive tissue inflammation seen in later stages of the disease.
